# Advantages of one step nucleic acid amplification (OSNA) whole node assay in sentinel lymph node (SLN) analysis in breast cancer

**DOI:** 10.1186/2193-1801-2-542

**Published:** 2013-10-17

**Authors:** Ana Santaballa, Helena De La Cueva, Carmen Salvador, Ana M García-Martínez, María J Guarín, David Lorente, Laura Palomar, Ismael Aznar, Fernando Dobón, Pilar Bello

**Affiliations:** Medical Oncology Department, Hospital Universitari i Politècnic La Fe, Valencia, Spain; Anatomic Pathology Department, Hospital Universitari i Politècnic La Fe, Valencia, Spain; Surgery Department, Hospital Universitari i Politècnic La Fe, Valencia, Spain; Nuclear Medicine Department, Hospital Universitari i Politècnic La Fe, Bulevar Sur s/n, 46026 Valencia, Spain

**Keywords:** Sentinel lymph node biopsy, Breast cancer, Molecular analysis, Micrometastases

## Abstract

**Background:**

The purpose of this study is to present our first results of sentinel node analysis (SLN) by one step nucleic acid amplification (OSNA) in routine clinical practice in our centre and compare them with the results of classic histopathological analysis in a historical cohort from our same institution.

**Methods:**

407 patients (total study population) with early breast cancer and no clinical nodal involvement underwent SLN biopsy in our institution. The SLN was analysed by OSNA in 164 biopsies. OSNA results were compared with the conventional histopathology study of 244 patients who had undergone SLN biopsy previously. The characteristics of the patients in both groups were evaluated and a comparison was made of the rate of metastases detected by both methods and of the surgical procedures needed in each group. We also investigated the state of non-sentinel lymph nodes if micrometastases where found in SLN.

**Results:**

SLN biopsy result was considered as positive in 45 patients (28%) in the OSNA group and in 58 in the historical group (24%). There was no difference in the rate of macrometastases (16,5% for OSNA, 20% for HE) but we found differences in the rate of micrometastases (11% for OSNA and 3,6% for HE p = 0.0007). Axillary lymphadenectomy (ALND) was performed in 43/45 cases in the OSNA group and in 51/58 of the historical group. In all patients diagnosed by OSNA, ALND was performed during the initial surgical procedure. In the historical cohort ALND was performed during the initial surgical procedure in 41 patients and in a second surgical procedure in 10 patients. Patients from both groups with micrometastases in the SLN had no metastases in other nodes when the ALND was performed.

**Conclusions:**

OSNA analysis allows the detection of SLN metastases as precisely as conventional pathology with an increased detection of micrometastases. The OSNA method can reduce the need of a deferred lymphadenectomy.

## Introduction

Selective sentinel node biopsy has replaced axillary lymph node dissection due to its lower morbidity and equivalent prognosis in the long-term (Giuliano et al. 1997; Krag et al. 2010). The presence of nodal metastases is an important prognostic factor and it can help in the asssessment of the need for adjuvant therapy after surgery. The conventional approach in the study for nodal metastases has been staining with haematoxylin-eosin (HE) and immunohistochemistry (IHC). This technique allows analysis during the primary breast tumour surgery, with some sections of the node analysed during the primary procedure, although the remaining sections have to be studied in a deferred manner, which increases the risk of losing valuable information during the surgical procedure.

The one-step acid amplification method (OSNA) detects and quantifies by polymerase chain reaction (PCR) the presence of mRNA of cytokeratin 19 in the whole node (Tsujimoto et al. 2007). It is more time-consuming than the HE study but it can be performed intraoperatively (Osako et al. 2011; Sagara et al. 2011).

The purpose of this study is to present our first results with the OSNA assay performed in a routine clinical setting in 163 patients with invasive and in situ breast cancer, examine their consistency with the classic HE study and evaluate the potential implications for the locorregional treatment of breast cancer patients.

## Patients and methods

### Patients

In the Hospital Universitari i Politècnic La Fe of Valencia 164 consecutive sentinel node biopsy procedures were performed in 163 patients with in situ and early breast cancer between March 2006 and December 2011, studied by OSNA technique. This analysis was performed intraoperatively and if metastases (both micro or macrometastases) were found axillary lymphadenectomy was practiced at the same time. This study has received the approval of the ethical local committee (Comité Ético de Investigación Científica del Hospital La Fe).

For comparison, 244 controls were used as a historical cohort; they were treated between March 2000 and April 2007 in the same hospital, and their sentinel node analysis was performed by the HE and immunohistochemical method.

In both groups, the criteria for sentinel node biopsy were: tumours up to 5 cm in size with no clinical involvement according to physical examination and ultrasound of the axilla. Table 1 shows the patient characteristics. Statistical analysis was performed with SPSS software, using the Chi square statistical test for associations between parameters of interest.

**Table 1:** **Patient characteristics**

Characteristics		OSNA cohort (n=164)	Historical cohort (n=244)
Age, median (range)		58 (33–86)	57 (30–93)
Pathologic tumour size, mean (range) mm		16.63 (0–60)	14.16 (0–55)
Tumour histology	DCIS	12	8
	Ductal invasive	131	192
	Lobular invasive	12	25
	Other subtypes invasive	8	19
SBR grade	1	51	100
	2	75	88
	3	34	17
	Unknown	3	39
Oestrogen receptor	Positive	142	143
	Negative	21	25
Progesterone receptor	Positive	125	202
	Negative	38	42
HER2 status	Positive	15	4
	Negative	139	26
	Unknown	9	214
Ki 67	≤ 10%	80	95
	> 10%	73	124
	Unknown	10	25

### Study of SLN by OSNA

After the fatty tissue was removed, the SLN was weighed and whole SLN was processed for the OSNA assay, which is based on the principles of the reverse transcription loop-mediated isothermal amplification method. The SLN was homogenized using LYNORHAG lysis buffer (Sysmex Corp., Hyogo, Japan). CK19 mRNA in each lysate was amplified using a LYNOAMP BC gene amplification reagent (Sysmex Corp.), then a 2-μl sample of each lysate was subjected to an RT-LAMP reaction. The CK19 mRNA copy number was detected by measuring the rise time based on a standard curve using an RD-100i (Sysmex Corp.).

The SLN was assessed as OSNA - when the CK 19 mRNA copy number was fewer than 2.5 × 10^2^ copies μl, OSNA + when it was between 2.5 × 10^2^ and 5.0 × 10^3^ copies μl, and OSNA ++ when it was more than 5.0 × 10^3^ copies μl. The OSNA assay is sometimes inhibited by inhibitory materials resulting in false negative (< 250 copies μl) reactions that may be resolved as positive (≥ 250 copies μl) reactions by simple dilution (1:10). However, the values of these reactions after dilution are less reliable for the quantitative assessment and were evaluated as + inhibition (+I).

### Study of sentinel node by IHC

The classical pathological study was based on a first-time intraoperative evaluation. The node was frozen and serial sections of the node, approximately 2 mm each, were performed. Histological staining of each section was done with haematoxylin-eosin. Subsequently, all complete node sections were fixed in paraffin and histological cuts were performed in intervals of 200 microns, which allowed for immunohistochemical staining with cytokeratin AE1-3 and Cam 5.2 and for the detection of isolated tumour cells.

To classify the findings in the lymph node the definition published in the seventh edition of the AJCC was used, which differentiates isolated tumour cells up to 0.2 mm in maximum diameter detected by immunohistochemistry [ITC or pN0 (i +)], tumour cells groups of more than 0.2 mm and up to 2 mm in diameter (micrometastases or pN1mi) and tumour cell groups with more than 2 mm (macrometastases or pN1a).

## Results

### Analysis of sentinel node

The incidence of lymph node metastases in the OSNA group was 45 (28%): 27 cases of macrometastases (17%) and 18 micrometastases (11%). In the conventional IHC control group any kind of metastases were found in 71 cases (29%): 49 patients showed macrometastases (20%), 9 micrometastases (4%), and 13 presented ITC (5%). Macrometastases rate differences were not statistically significant. Conversely, the difference in proportion of micrometastases is significant, with an increase of detection rate within the OSNA group (11 vs 4%, p = 0.007).

### Axillary lymphadenectomy in first vs. second time

Regarding the axillary surgery performed, all 18 OSNA patients who had micrometastases underwent axillary lymphadenectomy in the same surgical procedure, except in the case of a woman operated for bilateral breast cancer in whom there were micrometastases in the sentinel nodes of both sides and only a left axillary dissection was performed. In 26 of the 27 patients with macrometastases, a lymphadenectomy was performed during the initial surgery, while for one patient no further surgery was done.

Of the 49 women with macroscopically involved nodes in the historical cohort 41 underwent ALND at the same time as the SLN procedure while in the remaining 8 the axillary clearance was performed later, as the results were obtained after the operation. Of the 9 patients with micrometastases ALND was performed in a second time in two patients (these were two women whose primary tumour had to be re-excised due to close surgical margins); the other 7 did not undergo any other axillary surgical procedure.

### Analysis of non-sentinel lymph nodes in patients with micrometastases in the SLNB

After extending the number of axillary nodes analysed with axillary lymphadenectomy in patients with micrometastases in the SLN biopsy (2/9 patients in the historical cohort and 17/18 OSNA), there were no more affected lymph nodes outside the sentinel lymph node.

## Discussion

The sentinel node study by the OSNA method is being applied in an increasing number of hospitals. Its advantages over the traditional approach with haematoxylin-eosin and immunohistochemistry (HE/IHC) include the decrease in the workload for the pathologist as the process is largely automated and the ability to analyse the full node instead of just some sections while performing surgery of the primary breast tumour.

Different studies have shown that the OSNA assay has a good correlation with the pathological study (Tsujimoto et al. 2007; Sagara et al. 2011; Li et al.; Tamaki et al. 2009), and it also has a higher sensitivity for the detection of micrometastases (Osako et al. 2011).

In our series we have found a similar rate of macrometastases in SLN analysed by OSNA and HE/IHC but with an increased detection of micrometastases with OSNA.

However, this increase in the number of nodes with micrometastases provides information that is difficult to apply in the clinical setting, as its prognostic value has not been fully clarified. One of the most relevant studies to address this issue is the prospective NSABP B-32 trial, which was designed to evaluate the outcome of SLN biopsy alone compared to axillary dissection, in which 5,611 patients with SLN biopsy were randomized to undergo or not to undergo axillary clearance (Krag et al. 2007). Subsequently, an analysis was published of patients with occult nodal metastases reviewing almost 4000 ganglion blocks (Weaver et al. 2011). At five years, the presence of occult metastases was associated with a lower overall survival, progression free survival and distant metastases free interval compared to node-negative patients. Although this increased risk in the three parameters was statistically significant, the magnitude of the difference in the 5-year Kaplan-Meier estimates of overall survival was only 1.2 percentage points and the authors concluded that this difference might be of little clinical relevance.

The finding of micrometastases in the sentinel node can have an impact on clinical management. In our series all patients with micrometastases in the OSNA group underwent axillary clearance. One of the reasons why it was done was the study of a possible involvement of other lymph nodes, which is estimated to occur in approximately 10-15% of cases of SLN biopsy micrometastases in classical histological studies (Solá et al. 2013; Galimberti et al. 2013; Cserni et al. 2004; Houvenaeghel et al. 2009). In this sense, we cannot draw conclusions from our data since the rate of micrometastases in the HE/IHC group was very low and barely any lymphadenectomies were performed. Published data about the OSNA technique also indicate that the PCR load of cytokeratin 19 correlates with the finding of non-sentinel node metastases (Osako et al. 2011), although in our sample none of 17 patients undergoing lymphadenectomy due to micrometastasis in OSNA had involvement in the other nodes.

Despite the possible presence of other affected lymph nodes there are trials showing that lymphadenectomy can be omitted in patients with micrometastases in the SLN biopsy since their presence does not increase the rate of axillary recurrence (Solá et al. 2013; Galimberti et al. 2013; Guenther et al. 2003; Gant et al. 2003; Langer et al. 2005). Recently, two randomised trials that explore this issue had been published, a Spanish study (Solá et al. 2013) and the IBCSG 23–01 trial (Galimberti et al. 2013). With a recruitment lower than expected, 247 and 932 patients respectively with micrometastases in SLN biopsy were randomly assigned to axillary dissection or no axillary dissection. No differences in terms of disease-free survival after a follow-up of five years were seen.

Probably the long-term monitoring of patients in which micrometastases are detected by OSNA will provide new data in this sense, as it is a method that is more reproducible and allows a more complete analysis of the node than the histopathological analysis (Osako et al. 2011; Tamaki et al. 2009).

Currently in our centre micrometastases in SLN biopsy are not considered an indication for axillary clearance, given the growing literature that supports this decision and the recommendations in this sense of the 2011 St Gallen Consensus Conference (Goldhirsch et al. 2011). We maintain the indication in case of macrometastases although in the future this might be avoided in cases of macrometastases in only one or two sentinel nodes in certain patient, based on the findings of the ACOSOG Z0011 study (Giuliano et al. 2011).

In any case, the full analysis of lymph nodes by OSNA allows us to perform lymphadenectomy in the same surgical procedure in all cases, something not always possible in patients studied by IHC in which the full results can be obtained later. The performance of a single procedure can provide important advantages for the patient, such as avoiding a second operation and a possible delay in the start of adjuvant therapy, as well as lower cost for the institution (Guillen-Paredes et al. 2011).

## Conclusions

The sentinel node analysis by OSNA enables reliable results within minutes, allowing axillary lymphadenectomy practice in the same surgery when indicated, i.e. in case of SLN biopsy macrometastases.

The increase in diagnoses of patients with micrometastatic involvement by OSNA should be studied in terms of its prognostic and therapeutic implications.
